# Systematic Search for SARS-CoV-2 Main Protease Inhibitors for Drug Repurposing: Ethacrynic Acid as a Potential Drug

**DOI:** 10.3390/v13010106

**Published:** 2021-01-13

**Authors:** Camilla Isgrò, Anna Maria Sardanelli, Luigi Leonardo Palese

**Affiliations:** 1 Department of Basic Medical Sciences, Neurosciences and Sense Organs, University of Bari Aldo Moro, Piazza G. Cesare 11, 70124 Bari, Italy; camilla.isgro@uniba.it; 2 Department of Medicine, University Campus Bio-Medico of Rome, Via Alvaro del Portillo 21, 00128 Rome, Italy

**Keywords:** coronavirus, COVID-19, SARS-CoV-2, main protease, Mpro, 3C-like protease, 3CL protease, drug repurposing, protease inhibitors

## Abstract

In 2019 an outbreak occurred which resulted in a global pandemic. The causative agent has been identified in a virus belonging to the *Coronaviridae* family, similar to the agent of SARS, referred to as SARS-CoV-2. This epidemic spread rapidly globally with high morbidity and mortality. Although vaccine development is at a very advanced stage, there are currently no truly effective antiviral drugs to treat SARS-CoV-2 infection. In this study we present systematic and integrative antiviral drug repurposing effort aimed at identifying, among the drugs already authorized for clinical use, some active inhibitors of the SARS-CoV-2 main protease. The most important result of this analysis is the demonstration that ethacrynic acid, a powerful diuretic, is revealed to be an effective inhibitor of SARS-CoV-2 main protease. Even with all the necessary cautions, given the particular nature of this drug, these data can be the starting point for the development of an effective therapeutic strategy against SARS-CoV-2.

## 1. Introduction

In late 2019 a new pneumonia illness was first reported in Wuhan, China [1], named COVID-19 by the World Health Organization. It has rapidly spread over the world as pandemic threat with millions of infected and deaths. The causative agent of this pathology is a new betacoronavirus, related to the SARS coronavirus (SARS-CoV), designated as SARS-CoV-2 [2]. Coronaviruses (CoVs) [3,4] have large single-stranded, positive-sense RNA genomes (ranging from 25.5 to 32 kb). Several strains of CoVs are involved in pathological conditions in humans: particularly strains 229E, NL63, OC43, HKU1, MERS-CoV (Middle East Respiratory Sindrome), SARS-CoV (Severe Acute Respiratory Syndrome) and the recently appeared SARS-CoV-2. Structural proteins of CoVs are the spike (S) protein, membrane protein (M), envelop (E) protein and the nucleocapsid (N) protein. Some species contain also other structural proteins, such as the hemagglutinin esterase in beta-CoVs. The RNA genome contains several genes, whose order is generally preserved, coding for different proteins: PP1a, PP1ab, S, E, M, N. Two-third of the RNA genome is covered by the *ORF1a* and *ORF1b*, which produce two polyproteins, PP1a and PP1ab, whose processing leads to the formation of sixteen non-structural proteins (NSPs). NSPs participate in different viral functions, including the replicase-transcriptase complex. Two cysteine proteases in CoVs are involved in the specific cuts of these polyproteins to release the NSPs [5,6,7,8,9,10]. One is the papain-like protease ($PL^{pro}$), which performs three cleavage reactions. The other one is a chymotrypsin-like cysteine protease, known as main protease ($M^{pro}$) or 3C-like protease ($3CL^{pro}$) because of its similarity to the picornavirus 3C protease. $M^{pro}$ is responsible for the remaining 11 cuts leading to the formation of NSPs. The recognition sequence of $M^{pro}$ X-(L/F/M)-Q | (G/A/S)-X (where X is any amino acid and |represents the cleavage site) is not recognized by any host protease; consequently this enzyme represents an interesting target for the search of inhibitors as antiviral drugs in the treatment of CoV infections.

The $M^{pro}$ structure is similar in all CoV species [5]: this protease is a homodimer in which the N-terminus of one monomer participates to the substrate-specificity pocket and the oxyanion hole of the other monomer. Each monomer consists of two domains, I (residues 8-101 in 6LU7 [11]) and II (residues 102-184). The overall fold of these domains is chymotrypsin-like and harbor the enzyme catalytic site. A further α-helical domain (domain III, residues 201-303) is connected by a long loop to domain II and is involved in the dimerization. The shallow cleft between domains I and II contains a catalytic dyad, i.e., residues His 41 and Cys 145. These residues have an extremely conserved and rigid structural arrangement: the sulfur atom of Cys 145 is located at 3.6 Å from the N-ϵ of His 41. Moreover, it is interesting to note that a water molecule is generally visible at 3.2–3.3 Å from the N-δ of His 41 in crystals, thus suggesting that a catalytic *triad* could be at work in these enzymes [12].

This large body of knowledge, accumulated in a short time thanks to the enormous collective effort of the scientific community, on the structure and function of $M^{pro}$ has stimulated a number of works and methodology for in silico drug design [12,13,14,15,16]. Recently drug repositioning has been recognized as an alternative approach that explores new indications for approved (or also abandoned) drugs. Drug repositioning results in lower developmental expenses, since safety has been assessed and approved by regulatory authorities. Repurposing has been widely considered for the treatment of COVID-19 [17,18], including exploring new types of ligands or delivery systems [19,20]. Here we have evaluated the possibility of identifying inhibitors of this enzyme among molecules already used as drugs. The research was carried out both trying to discover reversible competitive inhibitors and inhibitors able not only to interact effectively with the active site but also to bind to it. None of the drugs approved for clinical use is capable of acting as a reversible competitive inhibitor of $M^{pro}$ with such efficiency to be considered for drug repurposing. However, our research has shown that ethacrynic acid is a potent irreversible inhibitor of the enzyme that could be further considered for the development of antiviral therapies.

## 2. Materials and Methods

The in silico analysis was conducted essentially as described [12,21,22,23]. Atomic coordinates of SARS-CoV-2 $M^{pro}$s were obtained from PDB [24]. The list considered in this work is reported as Table 1 [11,25,26,27,28,29,30,31,32,33,34,35,36]. For PCA and random projection analysis (RCA) a coarse-grained representation of the protein backbone was obtained considering the α-carbon atoms. Multiple conformations of the protein backbone were removed, and only the most represented conformation in the pdb file was retained. The α-carbon atoms of residues 1-304 were considered for the analyses. Structures were superposed by a Tcl script in a VMD [37] environment, as described [21,22,23]. The α-carbon atom Cartesian coordinates were extracted from the updated pdb files and stored in a data matrix, in which each row represented a $M^{pro}$ structure in the database. PCA was performed using the truncated SVD algorithm [38], which works even in the case of degenerate correlation matrices [21,22,23]; RCA was performed as described [39].

Molecular docking was performed using the AutoDock Vina software; *pdbqt* files were obtained by the same software or by the Open Babel toolbox [40,41]. Binding affinity was considered significant only for values lower than −6 kcal mol${}^{-1}$ [42]. The protein target *pdbqt* files were obtained by adding hydrogen atoms and charges were assigned using the Gasteiger method. Docking boxes were centered on the sulfur atom of Cys 145. The box dimensions were (28×32×34)Å and (28×26×34)Å for 5RET and 6LU7 respectively. Ligand structures has been obtained from ZINC and PubChem [43,44]. ZINC entries of the ligand data set are reported in Supplementary Materials, together with the binding affinity calculated on 5RET.

Activity assays were performed using the SARS-CoV-2 purified MBP-tagged $M^{pro}$ (BPS Bioscience) at a concentration value of 2 ng μL${}^{-1}$. The assays were carried out in the reaction buffer supplied by the manufacturer, in the presence of 3.18 μM of DTT deriving from the storage solution of the enzyme (DTT free condition) or in the presence of 1mM of DTT. Experiments were performed at room temperature in a Tecan microplate reader. An internally quenched fluorogenic FRET substrate (DABCYL-KTSAVLQSGFRKME-EDANS; BPS Bioscience) was used as substrate at a concentration value of 40 μM. For this peptide a $K_{m}$ of 17 μM and a $K_{cat}$ of 1.9 s${}^{-1}$ on the MBP-tagged $M^{pro}$ have been reported. GC376 (BPS Bioscience) was used at a concentration value of 100 μM as a negative control. The latter is an experimental veterinary drug [45,46] capable of inhibiting SARS-CoV-2 $M^{pro}$ with an $IC_{50}$ of approximately 0.46 μM.

## 3. Results

### 3.1. Protein Targets for Docking

**Figure 1:**
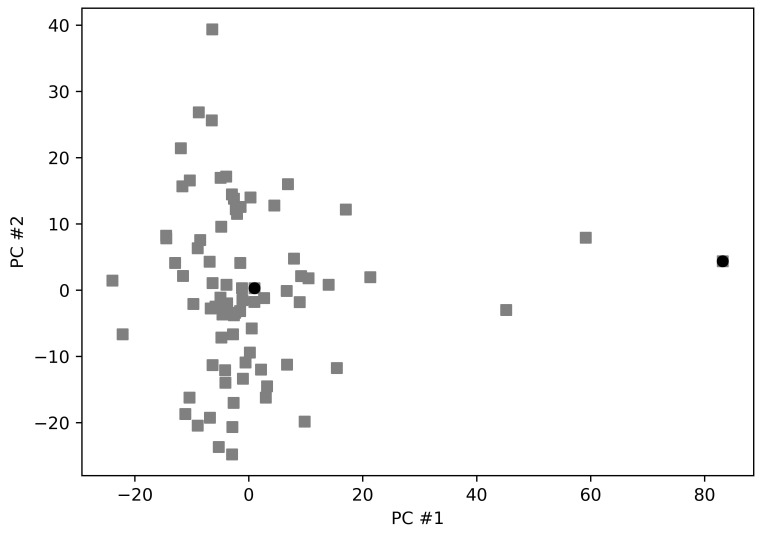
Principal component analysis of the $M^{pro}$ structures used in this work. Black circles represent the position in the plane identified by the first two principal components of 5RET and 6LU7.

Molecular docking is a method which predicts the mutual orientation of molecules when bound to each other to form a stable complex. This method can be used to predict the binding affinity of small ligands to a target protein by means of appropriate scoring functions. These (empirical) scoring functions contain a set of parameters which describe what are the most important properties in determining the binding affinity between the ligand and the receptor (i.e., the protein target). These scoring functions generally describe polar–apolar interactions, interaction entropy, desolvation effects, van der Waals interactions (by Lennard–Jones potential), and electrostatic interactions. Since the availability of fast scoring functions, molecular docking has become an important piece of the modern drug discovery toolbox. From a general point of view, it is necessary to pay particular attention to the strategy to be used for docking. The docking target must be a sufficiently representative structure: proteins are in fact extremely dynamic entities and this aspect must be taken into due account. In principle, protein dynamics can be considered by using a flexible target in the docking program, but this strategy exposes to the risk of over fitting, which leads to overestimate binding affinity. Our approach was therefore based on the use of different protein conformations such that the structural landscape of the protein of interest was sufficiently represented. Each of these representative conformations was then used to perform independent docking experiments in silico. A very efficient method to evaluate if different protein conformations are accessible to the protein of interest is to carry out the principal component analysis (PCA) on the protein structures available in the Protein Data Bank (PDB). Of course, this technique can only be used if a sufficient number of entries for the protein of interest are available. Fortunately, thanks to the international effort of structural biologists and crystallographers, an impressive number of SARS-CoV-2 $M^{pro}$ has been obtained. A detailed structural analysis on the conformational landscape of $M^{pro}$ based on a series of structures available in the PDB has already been reported elsewhere by one of the authors and will not be repeated in detail here [12]. We recall only the most interesting point for the purposes of the present work, namely that the $M^{pro}$ structures are grouped in a single cluster from which some outliers detach along the first principal component (Figure 1).

The root-mean-square deviation (RMSD) between the various structures is such that they are very similar to each other, so this data was also checked through the method of random projections. This latest analysis confirms that the outliers can be separated from the main group in two dimensions. Although the fold of the protein is overall preserved, as well as distances and orientation of catalytic residues in the active site, the binding site of the enzyme shows however some plasticity [47,48]. This last aspect of the protein structure is mainly due to side chain displacements and rotations, but with little involvement of the protein backbone [12]. Based on these results, we have chosen two structures as targets in molecular docking analyses. One of these was 5RET, as representative of the centroid of the distribution reported in Figure 1, while 6LU7 was chosen as the outlier representative. The structure 5RET is the $M^{pro}$ covalently linked to 1-4-[(3-chlorophenyl)methyl]piperazin-1-ylethan-1-one, whilst 6LU7 is blocked by ligand N3 [11,36]. From these structures the *pdbqt* files were prepared, after deleting the ligand, as described in Materials and Methods section.

### 3.2. Search for Competitive Inhibitors

In a first phase of the search for possible inhibitors of $M^{pro}$ we focused on potential competitive inhibitors. This target was carried out by analyzing a large number of molecules, i.e., essentially all FDA-approved molecules for which a structure file format for molecular docking was available in the ZINC database [44]. In this data set 2111 molecules are represented (see Supplementary Materials). In order to optimize the computation time, these molecules were first of all tested with the $M^{pro}$ structure reported as 5RET in the PDB (see Supplementary Materials). The molecules were then ranked according to the score obtained in the docking analysis on this target. Only those that have obtained a score corresponding to a binding energy of less than −7.5 kcal mol${}^{-1}$ were further analyzed using 6LU7 as a target. The rationale for this choice derives from the fact that a competitive inhibitor that can be used as a drug should have a binding affinity such as to give a sufficiently negative score on all the conformations of the target molecule. A total of 358 molecules were tested on 6LU7, and the mean of the binding affinity (on both conformations of the protease) was calculated. Finally, these molecules were ranked by ascending value of the average of the calculated binding energies. The drugs with the highest scores in molecular docking are reported in Table 2.

We tested the inhibitory activity on $M^{pro}$ of some of these substances in vitro, excluding those particularly toxic, such as anticancer drugs (it would be difficult to imagine antiblastic drugs as a therapy that can be administered in patients suffering from severe forms of COVID-19), or substances that act at very low concentrations on their known receptor to make them practically usable in repurposing for COVID-19 (e.g., ergotamine). We performed these experiments using purified $M^{pro}$ and an internally quenched substrate peptide, as described in the Materials and Methods section. The experiments were carried out by reading the fluorescence developed after the peptide cut at various incubation times (from 30 min to O.N.) in the presence of the various potential inhibitors at concentrations in the range 0.2–200 μM, and in the absence of inhibitors or in the presence of GC376 100 μM as positive and negative controls, respectively. Experiments were carried out in presence of dithiothreitol (DTT) in order to preserve the enzyme integrity in these long run experiments (see below Materials and Methods section). In absence of inhibitors the enzyme activity led to the development of an intense fluorescence, while in the presence of GC376 this was absolutely negligible. We tested some substances reported in Table 2 (Ciclesonide, Delafloxacin, Dutasteride, Netupitant, Tadalafil, Saquinavir) but no significant differences in fluorescence were observed with respect to the positive control (data not shown).

### 3.3. Search for Irreversible Inhibitors

Beside the search for potential competitive inhibitors described above, we conducted a search for irreversible inhibitors, able to covalently bind the active site of the SARS-CoV-2 $M^{pro}$. Since Cys 145 is the most accessible amino acid residue in the structure of the active site, our search has been limited to compounds capable of reacting with sulfhydryl groups, already approved as drugs for clinical use in humans. To this end, we re-analyzed the previously characterized compounds using binding affinity as inclusion criterion, and molecules exhibiting a binding affinity lower than −6 kcal mol${}^{-1}$ [42] were further evaluated. Several chemical groups are able to covalently bind (in a reversible or irreversible way) the sulfhydryl group of cysteine. Among the best known there are iodoacetamides and other haloacetamides, maleimides, disulfides, thiosulfates, acrylamide, α,β-unsaturated carbonyl compounds, α,β-unsaturated amides. The search for these reactive drug molecules was carried out on PubChem [43] using the appropriate SMILES code [49]. Molecules which fulfilled the aforementioned criteria on at least one protein conformation of the $M^{pro}$ were further analyzed in detail regarding the molecular docking poses. All the docking poses were manually inspected to evaluate the distance between the *warhead* of the drug and the sulfur atom of Cys 145 in the active site and their mutual orientation.

**Figure 2:**
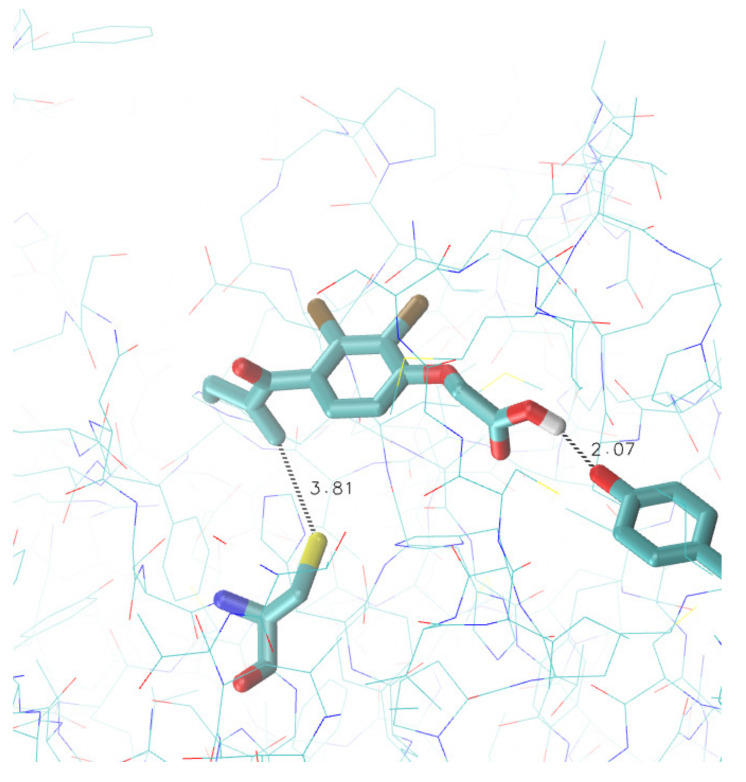
Molecular docking of ethacrynic acid at the active site of SARS-COV-2 $M^{pro}$. The protein structure correspond to the PDB entry 6LU7. Ethacrynic acid, Cys 145 and Tyr 54 are displayed in licorice. Reported distances are in Å.

These search criteria led to the identification of two compounds. One of these is boceprevir, with a binding affinity (regardless of the covalent bond) of −7.0 kcal mol${}^{-1}$. This compound has not been further analyzed because is a known inhibitor of $M^{pro}$ with an $IC_{50}$ = 8.0 μM [45]. The other compound identified in this study is the ethacrynic acid (IUPAC name: 2-[2,3-dichloro-4-(2-methylidenebutanoyl)phenoxy]acetic acid), an unsaturated ketone derivative of aryloxyacetic acid belonging to the class of loop diuretics. This molecule is able to bind to the active site of $M^{pro}$ with a calculated binding energy of approximately −6.0 kcal mol${}^{-1}$. Although the value of the bond energy is at the lower limit that we had imposed as significant for a specific bond, it must be considered that ethacrynic acid has a rather modest molecular weight (303.13 g mol${}^{-1}$). So, given the size of the molecule, the observed binding affinity value was suggestive of a specific interaction. In fact for a molecule of this size, in our set up, docking performed on randomly chosen regions of a randomly selected protein (a non-specific interaction) results in a calculated binding affinity approximately equal to (at most) −3.5 kcal mol${}^{-1}$. The analysis of the obtained poses revealed that the interaction between $M^{pro}$ and the ethacrynic acid is interesting. The α,β-unsaturated region of the molecule is often located at a distance of less than 4 Å from the sulfur atom of the Cys 145 in the active site of the enzyme. Moreover in these productive poses an interaction (hydrogen bond) between the carboxylic group of the ethacrynic acid and the aromatic hydroxyl group in Tyr 54 can be observed. This last residue participates in the formation of the active site. These interactions are shown in Figure 2.

**Figure 3:**
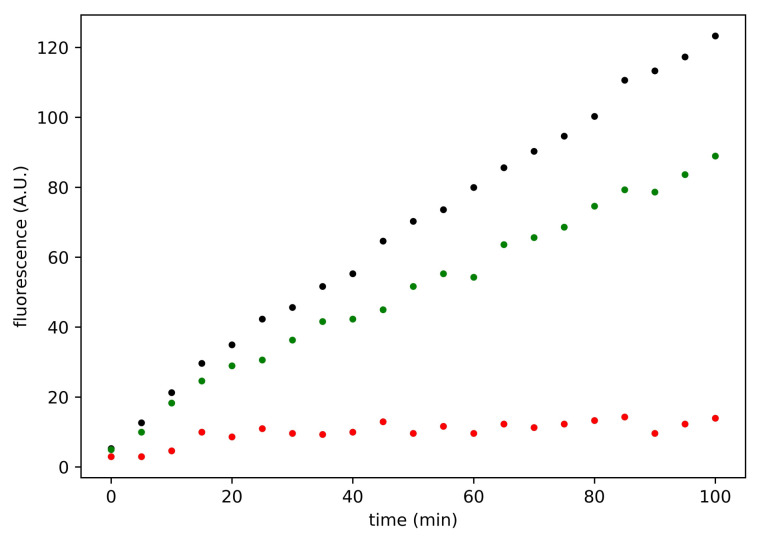
Time course of the SARS-CoV-2 $M^{pro}$ enzymatic activity at some inhibitor concentrations. Black, green and red dots represent positive control, 3.125 μM and 100 μM ethacrynic acid, respectively. Reported traces are the average of three experiments.

On the basis of these in silico evidences, we performed a series of in vitro assays to test the effective efficacy of the molecule in inhibiting the SARS-CoV-2 $M^{pro}$. These were conducted using the purified MBP-tagged enzyme as described in Materials and Methods section. The activity was evaluated in kinetic mode, using concentrations of enzyme and substrate such as to have zero-order kinetics (linear kinetics). The $V_{0}$ of the enzyme at various concentrations of ethacrynic acid was measured at least in triplicate. An example of the traces obtained in an experimental session is shown as Figure 3.

**Figure 4:**
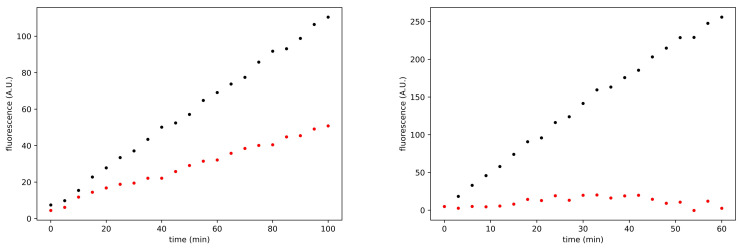
Time course of the SARS-CoV-2 $M^{pro}$ enzymatic activity. Left panel shows the enzyme activity in the DTT-containing reaction medium; black and red dots represent positive control and 100 μM ethacrynic acid, respectively. Right panel reports the enzyme activity in the DTT-free reaction medium; black and red dots represent positive control and 100 μM GC376, respectively. Reported traces are the average of three experiments.

The residual activity at the various concentrations of ethacrynic acid was obtained as the ratio between the $V_{0}$ of the inhibited enzyme and the $V_{0}$ of the enzyme in the absence of ethacrynic acid. The ethacrynic acid inhibitory action decreases in the presence of 1 mM DTT, and the residual $M^{pro}$ activity is around 40% at 100 μM of ethacrynic acid (see Figure 4, left panel).

**Figure 5:**
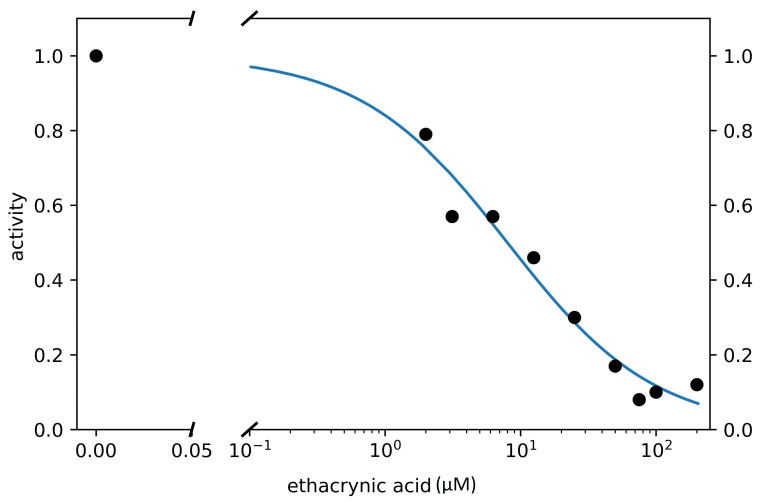
The ethacrynic acid inhibition assay on $M^{pro}$. Circles represent the mean of at least three independent replicates. Hill-type fitting curve described in the main text is also reported.

Using these data (DTT free conditions) we estimated the $EC_{50}$ of ethacrynic acid. The fitting with a Hill-type equation allows to estimate an $EC_{50}$ value of 8.0 μM (with a Hill coefficient of −0.8, that is slightly anticooperative). Figure 5 shows the results of this analysis. The estimate for the $EC_{50}$ obtained by considering a linear function in the semilogarithmic graph is equal to 9.5 μM. Both calculation methods therefore allow to estimate the $EC_{50}$ in the micromolar range under the experimental conditions used. These are extremely interesting values of the inhibition parameters, which place ethacrynic acid among the most potent $M^{pro}$ inhibitors among drugs approved for clinical use (compare the trace shown in Figure 3 obtained at 100 μM of ethacrynic acid with the same concentration of GC376 reported in Figure 4, right panel).

## 4. Discussion

This analysis highlights two very important aspects. The first, concerning the search for potential competitive inhibitors among drugs already approved for clinical use, it is that we have not been able to identify competitive inhibitors of SARS-CoV-2 $M^{pro}$ in this class of molecules. By molecular docking we obtained the binding affinity for all these substances (2111 in total). As can be observed by analyzing the scores reported in Table 2, unfortunately no drug approved for clinical use appeared to be a good candidate as competitive inhibitor of $M^{pro}$. Binding energy values, although high, were not very promising for the purpose of this study. As an example, the ergotamine re-docked on its receptor (PDB entry 4NC3) with our in silico set up showed a binding energy equal to −13.9 kcal mol${}^{-1}$, and nilotinib on the Bcr-Abl tyrosine kinase showed a binding energy equal to −11 kcal mol${}^{-1}$ [50,51]. Moreover, binding energies calculated for known pharmacological targets on the basis of the experimental $pK_{i}$ reported in the databases for these molecules suggested that, in any case, the effects on their pharmacological target would overtake any inhibitory activity on the SARS-CoV-2 protease. Anyway several of those with the highest score were tested in vitro to evaluate their effect on the activity of the enzyme. None of these have been shown to be a drug candidate for the treatment of COVID-19, as expected from the previous discussion. Even if we have not tested all the compounds listed in Table 2 for the reasons mentioned above (for example we have excluded anticancer drugs from the in vitro assays), even the highest value of binding affinity obtained is hardly compatible with that of a strong and specific competitive inhibitor. These results suggest that there are no competitive $M^{pro}$ inhibitors among the examined data set of drugs approved for human use. However, this does not exclude that these molecules may be effective through action on a different target (as shown in the analysis carried out in [52]).

The second important point is the finding of an interesting irreversible inhibitor, which could be considered for drug repurposing in the treatment of COVID-19. Interest in covalent drugs has been increasing in recent years, not only in the field of infectious disease treatment [53,54]. Alongside boceprivir, already described extensively in the literature [45], we have shown that a small molecule known for a long time in the clinical use, namely ethacrynic acid [55,56], binds effectively and irreversibly to SARS-CoV-2 $M^{pro}$. The inhibitory action is remarkable, as the $EC_{50}$ we have observed is in the micromolar range, comparable to that of boceprivir. In addition, not only the $EC_{50}$ value of ethacrynic acid is interesting, but also the residual activity of the $M^{pro}$ is noteworthy at very low values, comparable to the ones obtained in vitro using the best specific inhibitors currently known (for example boceprivir and GC376). Our results suggest that ethacrynic acid is a much more effective inhibitor of the SARS-CoV-2 $M^{pro}$ than the SARS-CoV homolog [57]. The 50% cytotoxic concentration in confluent cell monolayers of ethacrynic acid ranges between 84 μM and 173 μM in Vero and A549 cell lines respectively [58]. Moreover during intravenous infusion of 100 mg of ethacrynic acid, plasma concentrations increases to about 10 μg/mL [59]. This places ethacrynic acid among the best inhibitors of $M^{pro}$ in the class of molecules belonging to approved drugs and makes it a good candidate for drug repurposing.

A note of caution is required. The nature of the reactive group of the molecule is such that it can be bound by molecules containing reactive sulfhydryl groups. In vitro, in the presence of high concentrations of DTT the inhibitory action decreases. So, the efficacy of ethacrynic acid may be reduced in cells or tissues containing high concentrations of glutathione. But it must be considered that DTT is a compound that is able to reverse even the normal pharmacological effects of ethacrynic acid in cell [60], and this suggests that intracellular glutathione may not be sufficient to prevent the effects of this drug on the protease in infected cells. Moreover, ethacrynic acid is a powerful diuretic, which must be administered in a controlled manner to not exacerbate, for example, thrombotic phenomena as a consequence of strong alterations in fluid balance. Our hope is that these results will stimulate further research to evaluate the real efficacy of ethacrynic acid in treating COVID-19. Moreover, the fact that ethacrynic acid is able to inhibit the protease extremely effectively, could be the scaffold for medicinal chemistry studies to improve its efficiency.

## Figures and Tables

**Table 1:** PDB IDs of SARS-CoV-2 $M^{pro}$s.

5R7Y	5R7Z	5R80	5R81	5R82	5R83	5R84	5RE4	5RE5	5RE6
5RE7	5RE8	5RE9	5REA	5REB	5REC	5RED	5REE	5REF	5REG
5REH	5REI	5REJ	5REK	5REL	5REM	5REN	5REO	5REP	5RER
5RES	5RET	5REU	5REV	5REW	5REX	5REY	5REZ	5RF0	5RF1
5RF2	5RF3	5RF4	5RF5	5RF6	5RF7	5RF8	5RF9	5RFB	5RFC
5RFD	5RFE	5RFF	5RFG	5RFH	5RFI	5RFJ	5RFK	5RFL	5RFM
5RFN	5RFO	5RFP	5RFQ	5RFR	5RFS	5RFT	5RFU	5RFV	5RFW
5RFX	5RFY	5RFZ	5RG0	6LU7	6M03	6W63	6Y84		

**Table 2:** Highest score drugs obtained in molecular docking.

Drug	ZINC ID	Binding Affinity (kcal mol${}^{-1}$)
Dutasteride	ZINC3932831	−9.45
Ergotamine	ZINC52955754	−9.20
Dihydroergotamine	ZINC3978005	−9.15
Trametinib	ZINC43100709	−9.10
Nilotinib	ZINC6716957	−8.95
Bromocriptine	ZINC53683151	−8.85
Idarubicin	ZINC3920266	−8.80
Epirubicin	ZINC3938704	−8.80
Irinotecan	ZINC1612996	−8.80
Delafloxacin	ZINC3827556	−8.80
Naldemedine	ZINC100378061	−8.80
Lumacaftor	ZINC64033452	−8.75
Olaparib	ZINC40430143	−8.75
Daunorubicin	ZINC3917708	−8.75
Pimozide	ZINC4175630	−8.75
Palbociclib	ZINC3938686	−8.75
Teniposide	ZINC4099008	−8.75
Raltegravir	ZINC13831130	−8.70
Pazopanib	ZINC11617039	−8.65
Netupitant	ZINC11681563	−8.65
Saquinavir	ZINC26664090	−8.60
Eltrombopag	ZINC11679756	−8.60
Ciclesonide	ZINC3915154	−8.60
Midostaurin	ZINC100013130	−8.55
Azilsartan	ZINC14210642	−8.55
Tadalafil	ZINC3993855	−8.55
Vemurafenib	ZINC52509366	−8.50
Enasidenib	ZINC222731806	−8.50
Deferasirox	ZINC1481815	−8.50
Rolapitant	ZINC3816514	−8.50

## Data Availability

All data are reported in the text and Supplementary Materials.

## Supplementary Materials

The following are available at https://www.mdpi.com/1999-4915/13/1/106/s1, Table S1: The drug data set and bindingenergies on 5RET.

## References

[1] Wang C., Horby P.W., Hayden F.G., Gao G.F. (2020). A novel coronavirus outbreak of global health concern. Lancet.

[2] Gorbalenya A., Baker S., Baric R., de Groot R.J. (2020). The species Severe acute respiratory syndrome-related coronavirus: Classifying 2019-nCoV and naming it SARS-CoV-2. Nat. Microbiol..

[3] Lai M.M., Cavanagh D. (1997). The molecular biology of coronaviruses. Advances in Virus Research.

[4] Cui J., Li F., Shi Z.L. (2019). Origin and evolution of pathogenic coronaviruses. Nat. Rev. Microbiol..

[5] Hilgenfeld R. (2014). From SARS to MERS: Crystallographic studies on coronaviral proteases enable antiviral drug design. FEBS J..

[6] Anand K., Palm G.J., Mesters J.R., Siddell S.G., Ziebuhr J., Hilgenfeld R. (2002). Structure of coronavirus main proteinase reveals combination of a chymotrypsin fold with an extra *α*-helical domain. EMBO J..

[7] Anand K., Ziebuhr J., Wadhwani P., Mesters J.R., Hilgenfeld R. (2003). Coronavirus main proteinase (3CLpro) structure: Basis for design of anti-SARS drugs. Science.

[8] Wang F., Chen C., Tan W., Yang K., Yang H. (2016). Structure of main protease from human coronavirus NL63: Insights for wide spectrum anti-coronavirus drug design. Sci. Rep..

[9] Zhao Q., Li S., Xue F., Zou Y., Chen C., Bartlam M., Rao Z. (2008). Structure of the main protease from a global infectious human coronavirus, HCoV-HKU1. J. Virol..

[10] Ratia K., Saikatendu K.S., Santarsiero B.D., Barretto N., Baker S.C., Stevens R.C., Mesecar A.D. (2006). Severe acute respiratory syndrome coronavirus papain-like protease: Structure of a viral deubiquitinating enzyme. Proc. Natl. Acad. Sci. USA.

[11] Jin Z., Du X., Xu Y., Deng Y., Liu M., Zhao Y., Zhang B., Li X., Zhang L., Peng C. (2020). Structure of M pro from SARS-CoV-2 and discovery of its inhibitors. Nature.

[12] Palese L.L. (2020). The structural landscape of SARS-CoV-2 main protease: Hints for inhibitor search. ChemRxiv.

[13] Kapusta K., Kar S., Collins J.T., Franklin L.M., Kolodziejczyk W., Leszczynski J., Hill G.A. (2020). Protein reliability analysis and virtual screening of natural inhibitors for SARS-CoV-2 main protease (Mpro) through docking, molecular mechanic & dynamic, and ADMET profiling. J. Biomol. Struct. Dyn..

[14] Ojha P.K., Kar S., Krishna J.G., Roy K., Leszczynski J. (2020). Therapeutics for COVID-19: From computation to practices—Where we are, where we are heading to. Mol. Divers..

[15] Gimeno A., Mestres-Truyol J., Ojeda-Montes M.J., Macip G., Saldivar-Espinoza B., Cereto-Massagué A., Pujadas G., Garcia-Vallvé S. (2020). Prediction of Novel Inhibitors of the Main Protease (M-pro) of SARS-CoV-2 through Consensus Docking and Drug Reposition. Int. J. Mol. Sci..

[16] Muteeb G., Alshoaibi A., Aatif M., Rehman M.T., Qayyum M.Z. (2020). Screening marine algae metabolites as high-affinity inhibitors of SARS-CoV-2 main protease (3CLpro): An in silico analysis to identify novel drug candidates to combat COVID-19 pandemic. Appl. Biol. Chem..

[17] Li G., De Clercq E. (2020). Therapeutic options for the 2019 novel coronavirus (2019-nCoV). Nat. Rev. Drug Discov..

[18] Low Z.Y., Farouk I.A., Lal S.K. (2020). Drug repositioning: New approaches and future prospects for life-debilitating diseases and the COVID-19 pandemic outbreak. Viruses.

[19] Martorana A., Gentile C., Lauria A. (2020). In Silico Insights into the SARS CoV-2 Main Protease Suggest NADH Endogenous Defences in the Control of the Pandemic Coronavirus Infection. Viruses.

[20] Kumar S., Zhi K., Mukherji A., Gerth K. (2020). Repurposing antiviral protease inhibitors using extracellular vesicles for potential therapy of COVID-19. Viruses.

[21] Palese L.L. (2017). Conformations of the HIV-1 protease: A crystal structure data set analysis. Biochim. Biophys. Acta.

[22] Palese L.L. (2017). Analysis of the conformations of the HIV-1 protease from a large crystallographic data set. Data Brief.

[23] Gnoni A., De Nitto E., Scacco S., Santacroce L., Palese L.L. (2019). A New Look at the Structures of Old Sepsis Actors by Exploratory Data Analysis Tools. Antibiotics.

[24] Berman H.M., Westbrook J., Feng Z., Gilliland G., Bhat T.N., Weissig H., Shindyalov I.N., Bourne P.E. (2000). The Protein Data Bank. Nucleic Acids Res..

[25] Xue X., Yang H., Shen W., Zhao Q., Li J., Yang K., Chen C., Jin Y., Bartlam M., Rao Z. (2007). Production of authentic SARS-CoV Mpro with enhanced activity: Application as a novel tag-cleavage endopeptidase for protein overproduction. J. Mol. Biol..

[26] Yang S., Chen S.J., Hsu M.F., Wu J.D., Tseng C.T.K., Liu Y.F., Chen H.C., Kuo C.W., Wu C.S., Chang L.W. (2006). Synthesis, Crystal Structure, Structure- Activity Relationships, and Antiviral Activity of a Potent SARS Coronavirus 3CL Protease Inhibitor. J. Med. Chem..

[27] Zhu L., George S., Schmidt M.F., Al-Gharabli S.I., Rademann J., Hilgenfeld R. (2011). Peptide aldehyde inhibitors challenge the substrate specificity of the SARS-coronavirus main protease. Antiviral Res..

[28] Jacobs J., Grum-Tokars V., Zhou Y., Turlington M., Saldanha S.A., Chase P., Eggler A., Dawson E.S., Baez-Santos Y.M., Tomar S. (2013). Discovery, synthesis, and structure-based optimization of a series of N-(tert-butyl)-2-(N-arylamido)-2-(pyridin-3-yl) acetamides (ML188) as potent noncovalent small molecule inhibitors of the severe acute respiratory syndrome coronavirus (SARS-CoV) 3CL protease. J. Med. Chem..

[29] Yin J., Niu C., Cherney M.M., Zhang J., Huitema C., Eltis L.D., Vederas J.C., James M.N. (2007). A mechanistic view of enzyme inhibition and peptide hydrolysis in the active site of the SARS-CoV 3C-like peptidase. J. Mol. Biol..

[30] Verschueren K.H., Pumpor K., Anemüller S., Chen S., Mesters J.R., Hilgenfeld R. (2008). A structural view of the inactivation of the SARS coronavirus main proteinase by benzotriazole esters. Chem. Biol..

[31] Lee C.C., Kuo C.J., Hsu M.F., Liang P.H., Fang J.M., Shie J.J., Wang A.H.J. (2007). Structural basis of mercury-and zinc-conjugated complexes as SARS-CoV 3C-like protease inhibitors. FEBS Lett..

[32] Lee C.C., Kuo C.J., Ko T.P., Hsu M.F., Tsui Y.C., Chang S.C., Yang S., Chen S.J., Chen H.C., Hsu M.C. (2009). Structural basis of inhibition specificities of 3C and 3C-like proteases by zinc-coordinating and peptidomimetic compounds. J. Biol. Chem..

[33] Lu I.L., Mahindroo N., Liang P.H., Peng Y.H., Kuo C.J., Tsai K.C., Hsieh H.P., Chao Y.S., Wu S.Y. (2006). Structure-based drug design and structural biology study of novel nonpeptide inhibitors of severe acute respiratory syndrome coronavirus main protease. J. Med. Chem..

[34] Muramatsu T., Takemoto C., Kim Y.T., Wang H., Nishii W., Terada T., Shirouzu M., Yokoyama S. (2016). SARS-CoV 3CL protease cleaves its C-terminal autoprocessing site by novel subsite cooperativity. Proc. Natl. Acad. Sci. USA.

[35] Lee T.W., Cherney M.M., Huitema C., Liu J., James K.E., Powers J.C., Eltis L.D., James M.N. (2005). Crystal structures of the main peptidase from the SARS coronavirus inhibited by a substrate-like aza-peptide epoxide. J. Mol. Biol..

[36] Fearon D., Owen C., Douangamath A., Lukacik P., Powell A., Strain-Damerell C., Resnick E., Krojer T., Gehrtz P., Wild C. (2020). PanDDA Analysis Group Deposition of SARS-CoV-2 Main Protease Fragment Screen.

[37] Humphrey W., Dalke A., Schulten K. (1996). VMD: Visual molecular dynamics. J. Mol. Graph..

[38] Halko N., Martinsson P.G., Tropp J.A. (2011). Finding structure with randomness: Probabilistic algorithms for constructing approximate matrix decompositions. SIAM Rev..

[39] Palese L.L. (2018). A random version of principal component analysis in data clustering. Comput. Biol. Chem..

[40] Trott O., Olson A.J. (2010). AutoDock Vina: Improving the speed and accuracy of docking with a new scoring function, efficient optimization, and multithreading. J. Comput. Chem..

[41] O’Boyle N.M., Banck M., James C.A., Morley C., Vandermeersch T., Hutchison G.R. (2011). Open Babel: An open chemical toolbox. J. Cheminform..

[42] Shityakov S., Förster C. (2014). In silico predictive model to determine vector-mediated transport properties for the blood–brain barrier choline transporter. Adv. Appl. Bioinform. Chem..

[43] Kim S., Chen J., Cheng T., Gindulyte A., He J., He S., Li Q., Shoemaker B.A., Thiessen P.A., Yu B. (2019). PubChem 2019 update: Improved access to chemical data. Nucleic Acids Res..

[44] Sterling T., Irwin J.J. (2015). ZINC 15–ligand discovery for everyone. J. Chem. Inf. Model..

[45] Fu L., Ye F., Feng Y., Yu F., Wang Q., Wu Y., Zhao C., Sun H., Huang B., Niu P. (2020). Both Boceprevir and GC376 efficaciously inhibit SARS-CoV-2 by targeting its main protease. Nat. Commun..

[46] Vuong W., Khan M.B., Fischer C., Arutyunova E., Lamer T., Shields J., Saffran H.A., McKay R.T., van Belkum M.J., Joyce M. (2020). Feline coronavirus drug inhibits the main protease of SARS-CoV-2 and blocks virus replication. Nat. Commun..

[47] Fischer A., Sellner M., Neranjan S., Smieško M., Lill M.A. (2020). Potential Inhibitors for Novel Coronavirus Protease Identified by Virtual Screening of 606 Million Compounds. Int. J. Mol. Sci..

[48] Bzówka M., Mitusińska K., Raczyńska A., Samol A., Tuszyński J.A., Góra A. (2020). Structural and Evolutionary Analysis Indicate That the SARS-CoV-2 Mpro Is a Challenging Target for Small-Molecule Inhibitor Design. Int. J. Mol. Sci..

[49] Weininger D. (1988). SMILES, a chemical language and information system. 1. Introduction to methodology and encoding rules. J. Chem. Inf. Comput. Sci..

[50] Liu W., Wacker D., Gati C., Han G.W., James D., Wang D., Nelson G., Weierstall U., Katritch V., Barty A. (2013). Serial femtosecond crystallography of G protein–coupled receptors. Science.

[51] Weisberg E., Manley P.W., Breitenstein W., Brüggen J., Cowan-Jacob S.W., Ray A., Huntly B., Fabbro D., Fendrich G., Hall-Meyers E. (2005). Characterization of AMN107, a selective inhibitor of native and mutant Bcr-Abl. Cancer Cell.

[52] Tazikeh-Lemeski E., Moradi S., Raoufi R., Shahlaei M., Janlou M.A.M., Zolghadri S. (2020). Targeting SARS-COV-2 non-structural protein 16: A virtual drug repurposing study. J. Biomol. Struct. Dyn..

[53] Bauer R.A. (2015). Covalent inhibitors in drug discovery: From accidental discoveries to avoided liabilities and designed therapies. Drug Discov. Today.

[54] Singh J., Petter R.C., Baillie T.A., Whitty A. (2011). The resurgence of covalent drugs. Nat. Rev. Drug Discov..

[55] Melvin K.E.W., Farrelly R.O., North J.D.K. (1963). Ethacrynic Acid: A New Oral Diuretic. Br. Med. J..

[56] Molnar J., Somberg J.C. (2009). The clinical pharmacology of ethacrynic acid. Am. J. Ther..

[57] Kaeppler U., Stiefl N., Schiller M., Vicik R., Breuning A., Schmitz W., Rupprecht D., Schmuck C., Baumann K., Ziebuhr J. (2005). A new lead for nonpeptidic active-site-directed inhibitors of the severe acute respiratory syndrome coronavirus main protease discovered by a combination of screening and docking methods. J. Med. Chem..

[58] Smee D.F., Hurst B.L., Wong M.H. (2010). Lack of efficacy of aurintricarboxylic acid and ethacrynic acid against vaccinia virus respiratory infections in mice. Antivir. Chem. Chemother..

[59] Lacreta F.P., Brennan J.M., Nash S.L., Comis R.L., Tew K.D., O’Dwyer P.J. (1994). Pharmakokinetics and bioavailability study of ethacrynic acid as a modulator of drug resistance in patients with cancer. J. Pharmacol. Exp. Ther..

[60] Soltys B.J., Gupta R.S. (1994). Changes in mitochondrial shape and distribution induced by ethacrynic acid and the transient formation of a mitochondrial reticulum. J. Cell. Physiol..

